# Statewide trends and factors associated with genetic testing for hereditary cancer risk in Arkansas 2013–2018

**DOI:** 10.1186/s13053-022-00226-0

**Published:** 2022-05-23

**Authors:** Mahip Acharya, Kristin K. Zorn, Melinda E. Simonson, Milan Bimali, Gary W. Moore, Cheng Peng, Bradley C. Martin

**Affiliations:** 1 Division of Pharmaceutical Evaluation and Policy, Department of Pharmacy Practice, University of Arkansas for Medical Sciences College of Pharmacy, Education II Building 6253, 4301 W. Markham Street, slot 522, Little Rock, AR-72205 United States; 2 Department of Obstetrics and Gynecology, University of Arkansas for Medical Sciences College of Medicine, Little Rock, AR United States; 3 Department of Biostatistics, University of Arkansas for Medical Sciences, Little Rock, AR United States

**Keywords:** Hereditary cancers, Genetic testing, Arkansas, All Payer Claims Data, Predictive model

## Abstract

**Background:**

Early identification of hereditary cancer risk would save lives, but genetic testing (GT) has been inadequate. We assessed i) trends for hereditary breast and ovarian cancer (HBOC), Lynch syndrome, and other GT and ii) factors associated with receipt of GT.

**Methods:**

We used data from the Arkansas All-Payer Claims Database from January 2013 through June 2018 (commercial, Medicaid), December 2017 (state employee), or December 2016 (Medicare) and identified enrollees with ≥1 month of enrollment. Using Current Procedural Terminology (CPT-4) codes, rates for GT were calculated per 100,000 person-quarters and time series regressions estimated. Second, GT and covariate information for enrollees with 24 months of continuous enrollment were used to estimate separate logistic regression models for each GT category.

**Results:**

Among 2,520,575 unique enrollees, HBOC testing rates were 2.2 (Medicaid), 22.0 (commercial), 40.4 (state employee), and 13.1(Medicare) per 100,000 person-quarters and increased linearly across all plans. Older age (OR=1.24; 95%CI 1.20 – 1.28), female sex (OR=18.91; 95%CI 13.01 – 28.86), higher comorbidity burden (OR=1.08; 95%CI 1.05 – 1.12), mental disorders (OR=1.53; 95%CI 1.15 – 2.00), and state employee coverage (OR=1.65; 95%CI 1.37 – 1.97) were positively associated with HBOC testing. Less than 1 of 10,000 enrollees received Lynch syndrome testing, while < 5 of 10,000 received HBOC testing.

**Conclusion:**

GT rates for hereditary cancer syndromes have increased in Arkansas but remain low. Receipt of GT was explained with high discrimination by sex and plan type.

**Impact:**

Expansion of GT for hereditary cancer risk in Arkansas is needed to identify high-risk individuals who could benefit from risk-reduction strategies.

**Supplementary Information:**

The online version contains supplementary material available at 10.1186/s13053-022-00226-0.

## Introduction

Screening individuals unaffected by cancer for hereditary cancer syndromes (HCS) provides opportunities to reduce cancer risk by offering preventive strategies to those with elevated risk to mitigate or even eliminate cancer development. Significant advances in our understanding of the genes that underlie the most common HCS, hereditary breast and ovarian cancer (HBOC) and Lynch syndrome, have been made since the cloning of *BRCA1* in 1994, yet no more than 1%–10% of individuals at risk for these syndromes have been identified [1–3]. The landscape for genetic testing for HCS has evolved rapidly in the past decade [4]. Awareness of hereditary cancer risk has increased for the public and health care providers because of education efforts and disclosures of mutation status by celebrities such as Angelina Jolie [5]. Simultaneously, multiple companies now use next-generation sequencing platforms that allow for faster testing of large panels of genes at ever-decreasing cost [6, 7]. Against this national backdrop, Arkansas was the only Southeastern state to expand Medicaid in 2014 through a process referred to as the private option, which allowed approximately 350,000 people to gain insurance coverage in this relatively poor, rural state [8].

The need to expand utilization of genetic testing for HCS is clear, especially in the group of mutation carriers who are currently unaffected by cancer and therefore have the greatest potential to benefit from risk reduction. Data from the 2015 National Health Interview Survey (NHIS) indicate that around 2.5 million people have ever received cancer genetic testing, representing roughly 1% of the population [9]. Several groups were noted to have lower rates of genetic testing, including men, Hispanics, the uninsured, and those with less education [9]. Other data document that while rates of testing have increased modestly over the last decade, the increase has occurred mainly in commercially or publicly insured women with a family history of cancer [10]. Among women with a family history of breast or ovarian cancer, 4.2% of those with commercial insurance received genetic testing in 2015, up from 2.3% in 2010 [10]. Likewise, 2.8% of women with public insurance and a family history of breast or ovarian cancer received genetic testing in 2015, an increase from 0.3% in 2010 [10]. Others have observed considerable variation in testing by race and geographic location [11].

This study sought to describe the trends in genetic testing in a state with historically low levels of cancer genetic testing. The first objective was to assess the quarterly trends of genetic testing in four categories: HBOC testing, Lynch syndrome testing, tier 2 molecular pathology procedures (billing codes which include genes such as *TP53*, *VHL*, and *MLH1* based on the complexity of the testing procedure), and a composite measure of receipt of any cancer genetic testing, stratified by those with commercial, Medicaid, state employee, or Medicare coverage in Arkansas. The second objective was to identify demographic, clinical, and insurance coverage factors associated with enrollees receiving those four types of genetic testing.

## Methods

### Data

The Arkansas All-Payer Claims Database (APCD) is a state-level claims database that is harmonized across multiple payers and provides a nearly universal portrait of healthcare utilization of insured people in the state of Arkansas. It includes claims from commercial, Medicaid, state employee, and Medicare health plans for Arkansans. Data from January 2013 through June 2018 were used for commercial and Medicaid enrollees; due to lags in data availability, data from January 2013 through December 2017 were used for state employees and from January 2013 through December 2016 for Medicare enrollees. Medicare is the federal health insurance program for people who are 65 or older and younger persons with disabilities or end stage renal disease. Medicaid is a public insurance program for low-income families that is administered by states and jointly funded by the federal and state governments.

### Study sample

A separate sample set was constructed for each of the two objectives. For the trend analysis, enrollees with at least one month of enrollment in both medical and pharmacy benefits in each quarter were identified for each of the four insurance plan types. Enrollees were excluded from the sample if they had missing age or sex information. Enrollees who had overlapping enrollment in more than one plan type were only included in one plan using the following hierarchy: Medicare > Medicaid > state employee > commercial. For example, if an individual had overlapping enrollment in Medicare and Medicaid, they were only considered enrolled in Medicare. The total number of unique enrollees in each quarter for each plan type were retained as the denominator of persons who could have received genetic testing.

For the second objective, where exploratory models were estimated, individuals with 24 months of continuous enrollment in both medical and pharmacy benefits were identified for each of the four plan types. For individuals with more than 24 months of continuous enrollment, the most recent 24 months were considered. Individuals with more than one month of overlapping enrollment in two or more plan types were included in only one plan using the following hierarchy: state employee > commercial > Medicare > Medicaid. This hierarchy was adopted based on the empirical results for the rates of healthcare claims in the overlapping segments of the respective plans. The samples in the four plan types were combined into an overall sample. Individuals with missing age or sex were excluded. Enrollees who had a cancer diagnosis prior to genetic testing in the 24-month window and those less than 10 years of age at the start of the window were also excluded (Supplementary Figure 1). For example, an individual was considered to have received HBOC testing if they underwent testing prior to a cancer diagnosis in the 24-month study period. To avoid model overfitting, the overall sample was randomly divided into training and test sets in a 1:1 ratio. The models were developed using the training sets and evaluated using the test sets.

### Study measures

Genetic tests were identified from the inpatient and outpatient claims using Current Procedural Terminology (CPT-4) codes (Table 1). Tests were grouped into HBOC, Lynch syndrome, tier 2 molecular pathology procedures, other HCS panel testing , and any cancer genetic testing categories and counted in each quarter for each plan type. HBOC tests included tests for mutations in *BRCA1*, *BRCA2*, or a panel of genes including *BRCA1/2*. Similarly, Lynch syndrome tests included individual tests for the Lynch syndrome genes and panel tests including multiple genes. The category termed “tier 2 molecular pathology procedures” included billing codes that are based on the complexity of the testing technique, rather than referring to a specific gene or HCS; these are typically performed in low volumes for rare diseases (https://www.cms.gov/medicare-coverage-database/view/article.aspx?articleid=56199&ver=60&=). Unfortunately for this study’s purpose, these codes do not distinguish testing for genes related to cancer risk from those with non-cancer health risks; since important HCS genes such as TP53, VHL, and MLH1 are examples of tier 2 procedures, they were included in the analysis to avoid missing any cancer genetic testing. Additionally, a category called “other HCS panel testing” was used for HCS besides HBOC and Lynch syndrome that have specific billing codes, which included testing for hereditary polyposis/colorectal cancer risk, multiple endocrine neoplasia, and Cowden syndrome. Codes for broad test panels that included genes for multiple HCS were also included in the “other HCS” category. A separate analysis for the other HCS testing category was not conducted due to low prevalence; however, genetic tests for these syndromes were included in the composite “any cancer genetic test” category. Genetic tests in the HBOC, Lynch syndrome, tier 2 molecular pathology procedures, and other HCS panel testing categories were all included in the “any cancer genetic test” category. More than one inpatient or outpatient claim for genetic testing on the same day for an enrollee was counted as a single test. The quarterly rates for the genetic test measures were calculated for each quarter across four insurance plan types (commercial, state employee, Medicaid, and Medicare). Quarterly rates were also calculated for age- and sex-stratified groups: i) male enrollees <18 years old, ii) female enrollees <18 years old, iii) male enrollees 18–64 years old, iv) female enrollees 18–64 years old, v) male enrollees ≥65 years old, and vi) female enrollees ≥65 years old. Because of the different time periods for data across the four insurance plans, the age-sex stratified analyses were conducted using January 2013 through December 2016 data.

**Table 1:** Procedure codes used to identify genetic testing

Genetic testing categories	Description	CPT-4/HCPCS codes
HBOC syndrome testing	BRCA1/2	81162, 81163, 81164, 81165, 81166, 81167, 81211, 81212, 81213, 81214, 81215, 81216, 81217
	Panel	81432, 81433, 0102U, 0103U, 0129U, 0131U, 0132U, 0133U, 0138U
Lynch syndrome testing	Individual genes	81292, 81293, 81294, 81295, 81296, 81297, 81298, 81299, 81300, 81317, 81318, 81319
	Panel	0130U, 0162U
Other HCS panel testing	Hereditary polyposis genes	81201, 81202, 81203
	Multiple endocrine neoplasia panel	81437, 81438
	Cowden syndrome/PTEN	81321, 81322, 81323
	Hereditary multicancer panel	0104U, 0134U, 0135U, 81435, 81436
Tier 2 molecular pathology procedures^a^		81403, 81404, 81405, 81406, 81408, 81479

*HBOC* Hereditary Breast and Ovarian Cancer

*CPT-4* Current Procedural Terminology-4

*HCPCS* Healthcare Common Procedure Coding System

*HCS* Hereditary cancer syndrome

^a^ Tier 2 molecular pathology procedures are based on the complexity of the testing technique and are not specific to a gene or hereditary cancer syndrome.

For the second objective, separate binary measures were created for whether a subject received testing in any of the four genetic test categories and were used as the dependent variables in separate exploratory models. The factors explored were: age (calculated at the beginning of the 24-month enrollment segment), age squared, sex (recorded on the member enrollment file), mental health conditions recorded in inpatient or outpatient claims (anxiety disorders, developmental disorders, other miscellaneous mental disorders, nicotine dependence), health plan type, three-digit zip code region of residence in Arkansas (West=717, 718, 719; East=716, 720, 723; Central=721, 722; Northeast=724; North Central=725, 726; Northwest=727; Midwest=728, 729), and a measure of comorbidity (modified Elixhauser index). Age and age squared were included to account for a potential non-linear relationship between age and testing. Anxiety disorders, developmental disorders and other miscellaneous mental disorders were identified using ICD-9-CM and ICD-10-CM diagnosis codes. Nicotine dependence was identified using diagnosis codes or prescription claims for nicotine replacement therapy (Supplementary Table 2). The Elixhauser index was constructed using 31 comorbid conditions based on a published algorithm [12]. The existing Elixhauser index includes non-metastatic solid tumor and metastatic cancer conditions; these were removed for this study as the focus was enrollees who had genetic testing prior to a cancer diagnosis.

### Statistical analyses

Scatterplots were constructed to visualize the quarterly trends. Time series models were fit to estimate the overall trends using ordinary least squares (OLS) regressions with a first-order autoregressive error term. Linear and quadratic terms were used for the time (in quarters) variable. If the quadratic terms did not attain statistical significance, the models were re-estimated using only the linear term. For the exploratory models, logistic regression was used. Due to low test rates, Firth’s bias correction was applied. The predictive ability of the models were evaluated using c-statistic and Brier score for discrimination and calibration respectively. All analyses were conducted using SAS 9.4 and R 4.0.1. A two-sided p-value of 0.05 was used to determine statistical significance.

## Results

A total of 1,516,850 Medicaid, 1,501,828 commercial, 154,529 state employee, and 519,955 Medicare plan enrollees had at least one month of medical and pharmacy benefit enrollment in the aim 1 sample. The overall sample of unduplicated enrollees for the trend analysis was 2,520,575. The average age was 37 years; 53% were female (data not shown). For the second objective (exploratory models), a total of 1,495,960 individuals with 24 months of continuous medical and pharmacy enrollment were identified. The average age was 45 years; 56% were female (Table 2).

**Table 2:** Demographic, clinical and health plan characteristics of the sample used in the exploratory models (*n=*1,495,960)

Characteristics	N (%)
Age at first enrollment: mean (sd)	44.70 (22.60)
Elixhauser Comorbidity Index (Modified): mean (sd)	1.91 (2.80)
Sex	
Female	834,868 (55.81)
Male	661,092 (44.19)
Developmental Disorders	26,444 (1.77)
Anxiety Disorders	266,031 (17.78)
Other Mental Disorders	62,314 (4.17)
Nicotine Dependence Therapy	186,630 (12.48)
Plan type	
Commercial	560,177 (37.45)
State Employee	128,198 (8.57)
Medicaid	480,806 (32.14)
Medicare	326,779 (21.84)
Three-digit zip code regions	
West (717, 718, 719)	221,311 (14.79)
East (716, 720, 723)	377,101 (25.21)
Central (721, 722)	268,840 (17.97)
Northeast (724)	126,943 (8.49)
North Central (725, 726)	140,721 (9.41)
Northwest (727)	186,030 (12.44)
Midwest (728, 729)	175,014 (11.70)

### Trends in genetic testing

**Fig. 1:**
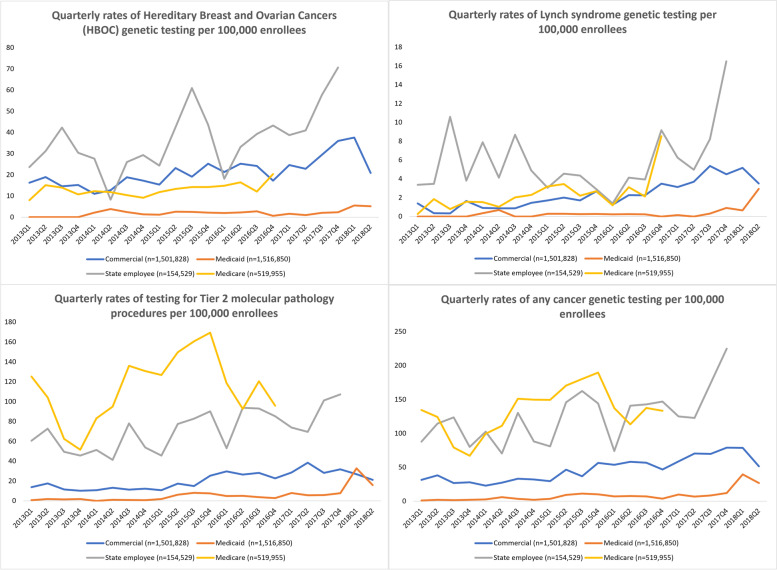
Quarterly Rates of HBOC, Lynch Syndrome, Tier 2 Molecular Pathology Procedures^*^, and Any^**^ Cancer Genetic Testing in Enrollees of Medicare, Commercial, Medicaid and State Employee Plans. HBOC – Hereditary Breast and Ovarian Cancer. * Tier 2 molecular pathology procedures are based on the complexity of the testing technique and are not specific to a gene or hereditary cancer syndrome. ** Any cancer genetic testing included tests in the HBOC, Lynch syndrome, Tier 2 molecular pathology procedures, and other hereditary cancer syndrome (HCS) panel categories

Figure 1 shows the quarterly trends of genetic testing for each of the four genetic testing categories for each of the four health insurance plan types. Over the vast majority of quarters, rates of HBOC testing were highest for state employee enrollees, followed by commercial, Medicare, and Medicaid enrollees. Lynch syndrome testing was the highest for state employee enrollees, followed by Medicare, commercial, and Medicaid enrollees. However, rates of tier 2 molecular pathology procedures were highest for Medicare, followed by state employee, commercial, and Medicaid enrollees. Rates for any cancer genetic testing (including HBOC, Lynch syndrome, other HCS panel testing, and tier 2 molecular pathology procedures) resembled those for tier 2 molecular pathology procedures.

HBOC, tier 2 molecular pathology procedures, and any cancer genetic testing increased linearly over time in Medicaid, commercial and state employee plans, as indicated by the significant positive linear trend terms in the time series models (p < 0.05; Table 3). The average quarterly increases in genetic testing for HBOC per 1 million person-quarters were greatest for state employee enrollees (16.2), followed by commercial (7.8), Medicare (3.5), and Medicaid (1.6) enrollees (Supplementary Figures 2-5). Testing for Lynch syndrome also increased linearly over time in commercial and Medicare enrollees (Supplementary Figures 6, 7), while it increased more dramatically toward the end of the time period in Medicaid and state employee plans, as evidenced by a significant positive quadratic term in the time series model (Table 3; Supplementary Figures 8, 9). Tier 2 molecular pathology procedures and any cancer genetic testing increased significantly over time for all types of insurance except for Medicare, where the increase was not significant (Table 3; Supplementary Figures 10-17).

**Table 3:** Time series coefficients and 95% confidence intervals of genetic testing trends by type of health plan coverage

	Commercial ***n=***1,501,828 β^**a**^ (95% CI)	Medicaid ***n=***1,516,850 β^**a**^ (95% CI)	State employee ***n=***154,529 β^**a**^ (95% CI)	Medicare ***n=***519,955 β^**a**^ (95% CI)
**HBOC testing**	0.78 (0.43, 1.13) *P-*value: <0.001	0.16 (0.02, 0.30) *P-*value: 0.024	1.62 (0.23, 3.02) *P-*value: 0.026	0.35 (0.07, 0.62) *P-*value: 0.020
**Lynch syndrome testing**	0.20 (0.14, 0.25) *P-*value: <0.001	-0.15 (-0.36, 0.06) *P-*value: 0.159 0.01 (0.00, 0.02)^b^ P-value: 0.045	-0.97 (-1.99, 0.05) P-value: 0.062 0.05 (0.01, 0.10)^b^ *P-*value: 0.028	0.21 (0.09, 0.33) *P-*value: 0.004
**Tier 2 molecular pathology procedures**^c^	0.89 (0.31, 1.47) *P-*value: 0.005	0.71 (0.26, 1.17) *P-*value: 0.004	2.34 (1.10, 3.58) *P-*value: 0.001	0.55 (-5.34, 6.43) *P-*value: 0.853
**Any cancer genetic testing**^d^	2.24 (1.46, 3.03) *P-*value: <0.001	0.99 (0.33, 1.66) *P-*value: 0.006	4.23 (1.53, 6.93) *P-*value: 0.004	1.82 (-3.93, 7.56) *P-*value: 0.520

*HBOC* Hereditary Breast and Ovarian Cancer

^a^ β represents the linear change per 100,000 persons per quarter

^b^ Quadratic trend term

^c^ Tier 2 molecular pathology procedures are based on the complexity of the testing technique and are not specific to a gene or hereditary cancer syndrome

^d^ Any cancer genetic testing included tests in the HBOC, Lynch syndrome, Tier 2 molecular pathology procedures, and other hereditary cancer syndrome (HCS) panel categories

**Fig. 2:**
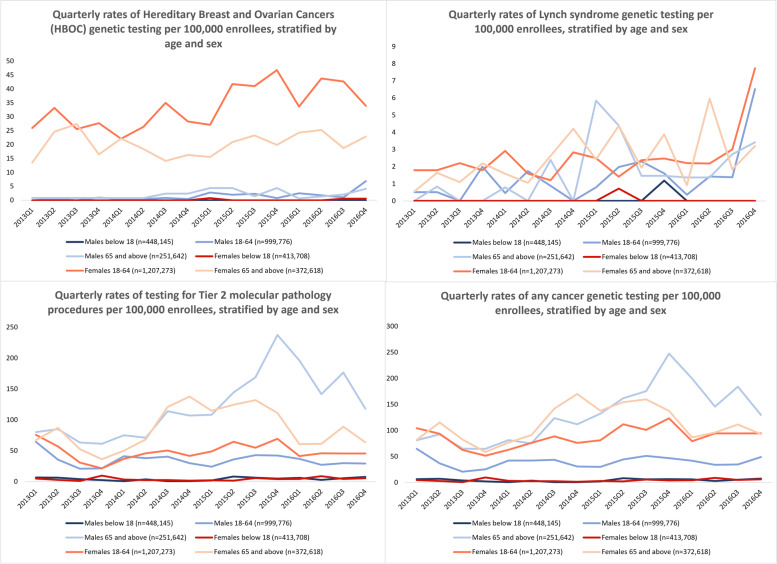
Quarterly Rates of HBOC, Lynch Syndrome, Tier 2 Molecular Pathology Procedures^*^, and Any^**^ Cancer Genetic Testing, Stratified by Age and Sex. HBOC – Hereditary Breast and Ovarian Cancer. * Tier 2 molecular pathology procedures are based on the complexity of the testing technique and are not specific to a gene or hereditary cancer syndrome. ** Any cancer genetic testing included tests in the HBOC, Lynch syndrome, Tier 2 molecular pathology procedures, and other hereditary cancer syndrome (HCS) panel categories

When analyzed by sex and age group, overall HBOC testing rates for male enrollees was lower than for female enrollees, with the highest rates among female enrollees aged 18-64 years (Fig. 2). Overall testing rates for Lynch syndrome were lower among men as well, with the highest rates seen in women >65 and 18-64 years old (Fig. 2). Among women, Lynch syndrome testing was much lower across age groups compared to HBOC testing, while rates for HBOC and Lynch syndrome were comparable for men. Because of the low rates of HBOC and Lynch syndrome testing among enrollees under age 18, time series models were not estimated. Testing for HBOC increased linearly over time for both men and women in the18–64-year-old age group, but not for either sex over age 64 (Table 4; Supplementary Figures 18-21). Lynch syndrome testing increased significantly over time for men aged 18-64 years and women ≥ 65 years (Table 4; Supplementary Figures 22-25). Tier 2 molecular pathology procedures and any cancer genetic testing increased linearly for male enrollees ≥65 years, with an average rate of increase of 66.8 per 1 million person-quarters but did not increase or decrease significantly for other groups (Table 4; Supplementary Figures 26-37).

**Table 4:** Time series coefficients for linear trend estimates and 95% confidence intervals of genetic testing trends by age and sex

	Male			Female		
	<18 Years ***n=***448,145 β* (95% CI)	18-64 Years ***n=***999,776 β^**a**^ (95% CI)	**>** 65 Years ***n=***251,642 β^**a**^ (95% CI)	<18 Years ***n=***413,708 β^**a**^ (95% CI)	18-64 Years ***n=***1,207,273 β^**a**^ (95% CI)	**>** 65 Years ***n=***372,618 β^**a**^ (95% CI)
**HBOC testing**	-	0.20 (0.11, 0.29) *P-*value: <0.001	0.16 (-0.02, 0.34) *P-*value: 0.074	-	1.12 (0.43, 1.82) *P-*value: 0.004	0.22 (-0.34, 0.78) *P-*value: 0.411
**Lynch syndrome testing**	-	0.16 (0.02, 0.31) *P-*value: 0.033	0.19 (-0.01, 0.39) *P-*value: 0.064	-	0.19 (-0.01, 0.38) *P-*value: 0.062	0.16 (0.07, 0.25) *P-*value: 0.003
**Tier 2 molecular pathology procedures**^**b**^	0.11 (-0.31, 0.54) *P-*value: 0.575	-0.91 (-2.69, -0.87) *P-*value: 0.289	6.68 (0.38, 12.98) ivalue: 0.039	0.13 (-0.16, 0.41) *P-*value: 0.350	-0.49 (-3.06, 2.08) *P-*value: 0.687	0.72 (-5.62, 7.06) *P-*value: 0.810
**Any cancer genetic testing**^**c**^	0.11 (-0.34, 0.57) *P-*value: 0.606	-0.06 (-1.86, 1.74) *P-*value: 0.946	7.35 (1.22, 13.47) *P-*value: 0.022	0.16 (-0.11, 0.42) *P-*value: 0.229	1.18 (-1.70, 4.07) *P-*value: 0.392	1.41 (-4.93, 7.76) *P-*value: 0.737

*HBOC* Hereditary Breast and Ovarian Cancer

^a^ β represents the linear change per 100,000 persons per quarter

^b^ Tier 2 molecular pathology procedures are based on the complexity of the testing technique and are not specific to a gene or hereditary cancer syndrome

^c^ Any cancer genetic testing included tests in the HBOC, Lynch syndrome, Tier 2 molecular pathology procedures, and other hereditary cancer syndrome (HCS) panel categories

### Factors associated with genetic testing

The exploratory models all had moderate to high levels of discrimination with c-statistics ≥ 0.79 (Table 5). Increasing age was associated with a higher likelihood of receiving all of the genetic testing examined. Female enrollees were much more likely to receive HBOC genetic testing than male enrollees (OR=18.91; 95% CI: 13.01–28.86) and were nearly twice as likely to receive testing for Lynch syndrome (OR=1.93; 95% CI: 1.11–3.51); however, women were slightly less likely to receive a tier 2 molecular pathology procedures genetic test (OR=0.90; 95% CI: 0.83–0.99) than men. Those with a higher comorbidity burden were more likely to receive all of the genetic tests examined. Relative to those with commercial health coverage, those with state employee coverage were more likely to receive all of the genetic tests examined, while those with Medicaid were less likely to receive HBOC, Lynch Syndrome, or any cancer genetic testing. Those with Medicare coverage also were less likely to receive HBOC or Lynch syndrome testing but were more likely to receive any cancer genetic test. Nicotine use and developmental disorders were not associated with receiving any of the genetic testing examined, while having an anxiety or other type of mental disorder were associated with a higher likelihood of receiving all of the genetic tests examined. The model results revealed significant geographic variation in receiving genetic testing. Relative to the Northwest part of Arkansas, receipt of any of the genetic tests examined was generally lower across all other regions except for receiving tier 2 molecular pathology procedures, which was higher for those living in the North-Central region of the state.

**Table 5:** Odds Ratios and 95% Confidence intervals for receiving one or more HBOC, lynch syndrome, tier 2 molecular pathology procedures, and any cancer genetic testing

Variable	HBOC Odds Ratio (95% CI)	HBOC (female-only sample) Odds Ratio (95% CI)	Lynch Syndrome Odds Ratio (95% CI)	General Genetic Testing^**a**^ Odds Ratio (95% CI)	Any Cancer Genetic Testing^b^ Odds Ratio (95% CI)
Age	1.24 (1.20, 1.28)	1.25 (1.21, 1.29)	1.22 (1.12, 1.36)	1.04 (1.03, 1.06)	1.06 (1.04, 1.07)
Age (Squared)	1.00 (1.00, 1.00)	1.00 (1.00, 1.00)	1.00 (1.00, 1.00)	1.00 (1.00, 1.00)	1.00 (1.00, 1.00)
Modified Elixhauser Index	1.08 (1.05, 1.12)	1.07 (1.03, 1.10)	1.12 (1.03, 1.20)	1.13 (1.12, 1.14)	1.13 (1.12, 1.14)
Sex (reference = Male) Female	18.91 (13.01, 28.86)	-------	1.93 (1.11, 3.51)	0.90 (0.83, 0.99)	1.22 (1.12, 1.32)
Health Plan (reference = Commercial)					
State employee	1.65 (1.37, 1.97)	1.64 (1.36, 1.97)	1.66 (0.77, 3.34)	4.54 (3.94, 5.23)	2.66 (2.35, 3.00)
Medicaid	0.24 (0.18, 0.32)	0.22 (0.17, 0.30)	0.78 (0.36, 1.58)	0.52 (0.43, 0.64)	0.33 (0.28, 0.40)
Medicare	0.41 (0.30, 0.56)	0.38 (0.27, 0.52)	0.86 (0.41, 1.74)	3.93 (3.45, 4.50)	2.42 (2.16, 2.71)
State Region (reference = Northwest)		0.43 (0.31, 0.57)	0.63 (0.26, 1.53)	0.85 (0.72, 1.01)	0.79 (0.68, 0.91)
West	0.42 (0.31, 0.56)				
East	0.70 (0.57, 0.88)	0.69 (0.56, 0.87)	0.62 (0.28, 1.39)	0.83 (0.71, 0.97)	0.83 (0.72, 0.95)
Central	0.66 (0.52, 0.84)	0.66 (0.52, 0.84)	0.37 (0.13, 0.93)	1.11 (0.95, 1.29)	1.01 (0.88, 1.17)
Northeast	0.28 (0.15, 0.36)	0.23 (0.14, 0.36)	0.30 (0.06, 1.05)	0.72 (0.58, 0.88)	0.64 (0.53, 0.77)
North Central	0.59 (0.43, 0.80)	0.56 (0.40, 0.77)	1.47 (0.66, 3.38)	0.51 (0.41, 0.63)	0.54 (0.45, 0.65)
Midwest	0.42 (0.30, 0.57)	0.41 (0.30, 0.57)	0.69 (0.26, 1.76)	1.20 (1.02, 1.42)	1.07 (0.92, 1.27)
Any Nicotine Usage (reference = No)	0.88 (0.70, 1.11)	0.90 (0.71, 1.13)	1.05 (0.54, 1.95)	1.07 (0.95, 1.20)	1.01 (0.90, 1.12)
Developmental Disorders (reference = No)	0.85 (0.28, 1.92)	0.97 (0.32, 2.20)	0.43 (0.00, 3.09)	1.11 (0.84, 1.44)	1.12 (0.86, 1.44)
Anxiety Disorders (referent = No)	1.19 (0.99, 1.43)	1.22 (1.01, 1.47)	1.37 (0.75, 2.45)	1.61 (1.45, 1.78)	1.54 (1.40, 1.67)
Other Mental Disorders (reference = No)	1.53 (1.15, 2.00)	1.56 (1.17, 2.05)	4.36 (2.28, 7.98)	1.60 (1.39, 1.83)	1.59 (1.40, 1.80)
Model c-statistic	0.88	0.81	0.82	0.82	0.79
Model Brier score	<0.001	<0.001	<0.001	0.003	0.003

*HBOC* Hereditary Breast and Ovarian Cancer

^a^ Tier 2 molecular pathology procedures are based on the complexity of the testing technique and are not specific to a gene or hereditary cancer syndrome

^b^ Any cancer genetic testing included tests in the HBOC, Lynch syndrome, Tier 2 molecular pathology procedures, and other hereditary cancer syndrome (HCS) panel categories

## Discussion

This analysis of APCD data shows that genetic testing for hereditary cancer susceptibility increased moderately in a linear fashion from 2013 through 2018 in Arkansas. Applying the time series coefficients to our baseline rates of testing suggests that testing for HBOC and Lynch syndrome nearly doubled for those with commercial and state employee coverage. In comparison, Knerr *et al.* reported a 33% increase in *BRCA* testing from 2005 to 2015 in women over 18 without an incident breast or ovarian cancer within their hospital system in Washington state [13]. Pace *et al.* reported that in Massachusetts, the mean monthly number of tests per 100,000 women doubled to quadrupled from 2011 to 2015, depending on the type of insurance [14]. However, the absolute rates of testing for HCS in Arkansas remained low. Less than 1 out of every 10,000 persons received Lynch syndrome testing, while fewer than 5 out of every 10,000 received HBOC testing. Estimates for the prevalence of mutations in the general US population are 1/300 for Lynch syndrome and 1/400 for *BRCA1/2*, emphasizing that many mutation carriers remain undiagnosed [1–3].

Additionally, our data suggest that health plan coverage is an important determinant in accessing cancer genetic testing. State employees had the highest rates of HBOC and Lynch syndrome testing and equally high rates as Medicare enrollees for any cancer genetic testing. Medicare enrollees had the highest rate for tier 2 molecular pathology procedures. The testing rates were substantially lower for Medicaid enrollees compared to enrollees in commercial, state employee, and Medicare plans. The overall rates of HBOC and Lynch syndrome testing were highest among women 18–64 years old, while rates of tier 2 molecular pathology procedures and any cancer genetic testing were highest among men 18–64 years old. In multivariate exploratory models that controlled for key demographic differences, those with state employee coverage were 1.6 to 4.5 times more likely to receive genetic testing compared to commercial enrollees, while commercial enrollees were substantially more likely to receive any type of genetic testing than Medicaid enrollees. Type of coverage played a similarly important role in a study more narrowly focused on women receiving BRCA1/2 testing in Massachusetts; testing among privately insured women increased from 9.3 in 2011 to 18.4 in 2015, while among Medicaid enrollees, it increased from 3.7 in 2011 to 14.7 in 2015 [14].

The lower rates of genetic testing in Medicaid enrollees may be due to several factors. First, 51% of all Arkansas Medicaid recipients in 2017 were age 20 or younger, the age group that is the least likely to have cancer genetic testing [15]. However, multivariate models controlling for age found that Medicaid recipients remained the least likely to receive any of the genetic testing examined, which suggests that substantial disparities may exist in the diagnosis of hereditary cancers across economic classes. The Affordable Care Act (ACA) implemented in 2014 mandated that genetic testing for HBOC be considered a preventive service in qualified individuals and be covered without cost sharing [15]. Arkansas participated in ACA expansion of access to insurance, unlike many other Southern states; however, this participation did not yield increases in receipt of genetic testing for Medicaid enrollees comparable to those with state employee or commercial coverage. Many studies document that physicians are less likely to discuss and order genetic screening among racial/ethnic minorities and individuals with less than a college level education [9, 16–18].

When genetic testing is offered by the physician, the cost is often mentioned as a major hurdle, with 49% of persons considering out-of-pocket cost as a major deterrent [19]. Some companies providing testing have specific policies that preclude billing patients for testing if Medicaid does not cover the test, but not all providers offering testing know the details of these financial policies for each company. Our results echo other data that document only modest increases in genetic testing since the ACA was implemented, suggesting that this provision may have expanded access to these services but may not be sufficient to reach all those at risk for hereditary cancer [20].

Considerable variation exists among commercial payers regarding coverage for genetic testing, which could partially explain the low uptake of testing in our study. A 2015 study reported that 76% of the private payers had coverage policies for *BRCA1/2* testing [21]. The coverage for multigene panel testing was low, with only 23% of the payers covering panel testing and only under the condition that all genes tested in the panel were medically necessary for the individuals [22, 23]. One explanation provided by many commercial payers is that they deem multigene testing an experimental diagnostic or treatment approach [21, 23, 24]. Additionally, 70% of private payers required prior authorization for both single and multigene testing in a 2018 study [23]. High-quality studies demonstrating penetrance and effectiveness of multigene panels in a clearly defined population could persuade private payers to cover panel testing and likely increase its utilization; obtaining such data, though, is complicated by the differences in the genes included in panels offered by different companies. In the meantime, companies providing genetic testing for HCS have coped with the lack of coverage of gene panels by billing for testing for specific genes, such as *BRCA1/2*, that are routinely covered by insurance rather than all the genes included on the panel.

Women had 18-fold higher odds of testing for HBOC and had nearly 2-fold higher odds of testing for Lynch syndrome, which confirms a consistent finding that men are less likely to receive genetic testing than women [9]. Anxiety disorders and miscellaneous mental health disorders (which included conditions such as sleep and dissociative disorders) had positive associations with general genetic testing and HBOC testing in women. A previous study in women from *BRCA1/2* mutation-negative families reported that women who had a higher perceived lifetime risk of cancer and higher worry about cancer were more likely to show an interest in genetic testing [25]. Another study, however, reported no association between distress and participation in *BRCA* testing [26]. Given the cross-sectional approach in our study, we cannot comment on whether the positive association between anxiety and other mental health disorders and genetic testing is due to mental health diagnoses before or after genetic testing.

This study has several limitations. We could not evaluate genetic testing trends across racial and ethnic groups. Unfortunately, the claims data for commercial insurance lack information on race and ethnicity. We did not include enrollees with a personal history of cancer in the current analysis; our analysis of genetic testing trends is focused on those who had not had cancer. Further, claims data reflect that a genetic test was performed, not the result of the test; we know that an enrollee had a test for HBOC, for example, but not whether that test documented a mutation. Insurance plan types have different lags in data availability, limiting our ability to evaluate the trends over a consistent time period for all four plan types. The exploratory models used a combination of cross-sectional and longitudinal approaches, the results of which should be interpreted as exploratory associations. Finally, the structure of billing codes for genetic testing for HCS is complex, constantly evolving, and does not allow for specific designations of each gene included in a testing panel at this time. The tier 2 molecular molecular proceduresrefer to the complexity of the testing procedure, not the genes tested, resulting in codes that include both cancer and non-cancer testing. Consequently, our ‘any cancer genetic’ test category, which includes tier 2 molecular tests, likely overstates genetic testing specific to cancer.

This study documented a modest increase in genetic testing for HBOC and Lynch syndrome across Medicaid, commercial, state employee and Medicare plans in Arkansas, although rates were lower than that observed in other states. The exploratory models highlight the influence of sex and health care coverage as a key determinant in accessing genetic screening. Further studies are ongoing to understand the barriers to genetic testing in Arkansas.

## Supplementary Information

**Additional File 1: (Supplementary Table 1and Supplementary Figures 1 – 37). Supplementary Table 1.** Diagnosis codes used to identify mental health disorders. **Supplementary Figure 1.** Sample selection for the exploratory models (2nd objective of the study). **Supplementary Figure 2.** Actual and predicted rates of Hereditary Breast and Ovarian Cancer (HBOC) genetic testing in Medicaid enrollees. **Supplementary Figure 3.** Actual and predicted rates of Hereditary Breast and Ovarian Cancer (HBOC) genetic testing in Commercial plan enrollees. **Supplementary Figure 4.** Actual and predicted rates of Hereditary Breast and Ovarian Cancer (HBOC) genetic testing in State Employee plan enrollees. **Supplementary Figure 5.** Actual and predicted rates of Hereditary Breast and Ovarian Cancer (HBOC) genetic testing in Medicare enrollees. **Supplementary Figure 6.** Actual and predicted rates of Lynch syndrome genetic testing in Medicaid enrollees. **Supplementary Figure 7.** Actual and predicted rates of Lynch syndrome genetic testing in Commercial enrollees. **Supplementary Figure 8.** Actual and predicted rates of Lynch syndrome genetic testing in State employee plan enrollees. **Supplementary Figure 9.** Actual and predicted rates of Lynch syndrome genetic testing in Medicare enrollees. **Supplementary Figure 10.** Actual and predicted rates of Tier 2 molecular pathology procedures* in Medicaid enrollees. **Supplementary Figure 11.** Actual and predicted rates of Tier 2 molecular pathology procedures* in Commercial plan enrollees. **Supplementary Figure 12.** Actual and predicted rates of Tier 2 molecular pathology procedures* in State employee plan enrollees. **Supplementary Figure 13.** Actual and predicted rates of Tier 2 molecular pathology procedures* in Medicare enrollees. **Supplementary Figure 14.** Actual and predicted rates of any cancer genetic testing* in Medicaid enrollees. **Supplementary Figure 15.** Actual and predicted rates of any cancer genetic testing* in Commercial plan enrollees. **Supplementary Figure 16.** Actual and predicted rates of any cancer genetic testing* in State employee plan enrollees. **Supplementary Figure 17.** Actual and predicted rates of any cancer genetic testing* in Medicare enrollees. **Supplementary Figure 18.** Actual and predicted rates of Hereditary Breast and Ovarian Cancer (HBOC) genetic testing in men 18–64 years old. **Supplementary Figure 19.** Actual and predicted rates of Hereditary Breast and Ovarian Cancer (HBOC) genetic testing in men ≥65 years old. **Supplementary Figure 20.** Actual and predicted rates of Hereditary Breast and Ovarian Cancer (HBOC) genetic testing in women 18–64 years old. **Supplementary Figure 21.** Actual and predicted rates of Hereditary Breast and Ovarian Cancer (HBOC) genetic testing in women ≥65 years old. **Supplementary Figure 22.** Actual and predicted rates of Lynch syndrome genetic testing in men 18–64 years old. **Supplementary Figure 23.** Actual and predicted rates of Lynch syndrome genetic testing in men ≥65 years old. **Supplementary Figure 24.** Actual and predicted rates of Lynch syndrome genetic testing in women 18–64 years old. **Supplementary Figure 25.** Actual and predicted rates of Lynch syndrome genetic testing in women ≥65 years old. **Supplementary Figure 26.** Actual and predicted rates of Tier 2 molecular pathology procedures* in male enrollees <18 years old. **Supplementary Figure 27.** Actual and predicted rates of Tier 2 molecular pathology procedures* in men 18–64 years old. **Supplementary Figure 28.** Actual and predicted rates of Tier 2 molecular pathology procedures* in men ≥65 years old. **Supplementary Figure 29.** Actual and predicted rates Tier 2 molecular pathology procedures* in female enrollees <18 years old. **Supplementary Figure 30.** Actual and predicted rates of Tier 2 molecular pathology procedures* in women 18–64 years old. **Supplementary Figure 31.** Actual and predicted rates of Tier 2 molecular pathology procedures* in women ≥65 years old. **Supplementary Figure 32.** Actual and predicted rates of any cancer genetic testing* in male enrollees <18 years old. **Supplementary Figure 33.** Actual and predicted rates of any cancer genetic testing* in men 18–64 years old. **Supplementary Figure 34.** Actual and predicted rates of any cancer genetic testing* in men ≥65 years old. **Supplementary Figure 35.** Actual and predicted rates of any cancer genetic testing* in female enrollees <18 years old. **Supplementary Figure 36.** Actual and predicted rates of any cancer genetic testing* in women 18–64 years old. **Supplementary Figure 37.** Actual and predicted rates of any cancer genetic testing* in women.

## Data Availability

The data that support the findings of this study are available from the Arkansas Center for Health Care Improvement, but restrictions apply to the availability of these data, which were used under license for the current study, and so are not publicly available. The original data are however available from the Arkansas Center for Health Care Improvement authors upon reasonable request and with permission of [third party name].
