# Supplementary material for: Statewide trends and factors associated with genetic testing for hereditary cancer risk in Arkansas 2013–2018
Source: Hered Cancer Clin Pract. 2022 May 23;20:19. doi: 10.1186/s13053-022-00226-0 (PMC9128197; doi:10.1186/s13053-022-00226-0)
Supplement: Supplementary file 1 — Additional File 1: (Supplementary Table 1and Supplementary Figures 1 – 37). Supplementary Table 1. Diagnosis codes used to identify mental health disorders. Supplementary Figure 1. Sample selection for the exploratory models (2nd objective of the study). Supplementary Figure 2. Actual and predicted rates of Hereditary Breast and Ovarian Cancer (HBOC) genetic testing in Medicaid enrollees. Supplementary Figure 3. Actual and predicted rates of Hereditary Breast and Ovarian Cancer (HBOC) genetic testing in Commercial plan enrollees. Supplementary Figure 4. Actual and predicted rates of Hereditary Breast and Ovarian Cancer (HBOC) genetic testing in State Employee plan enrollees. Supplementary Figure 5. Actual and predicted rates of Hereditary Breast and Ovarian Cancer (HBOC) genetic testing in Medicare enrollees. Supplementary Figure 6. Actual and predicted rates of Lynch syndrome genetic testing in Medicaid enrollees. Supplementary Figure 7. Actual and predicted rates of Lynch syndrome genetic testing in Commercial enrollees. Supplementary Figure 8. Actual and predicted rates of Lynch syndrome genetic testing in State employee plan enrollees. Supplementary Figure 9. Actual and predicted rates of Lynch syndrome genetic testing in Medicare enrollees. Supplementary Figure 10. Actual and predicted rates of Tier 2 molecular pathology procedures* in Medicaid enrollees. Supplementary Figure 11. Actual and predicted rates of Tier 2 molecular pathology procedures* in Commercial plan enrollees. Supplementary Figure 12. Actual and predicted rates of Tier 2 molecular pathology procedures* in State employee plan enrollees. Supplementary Figure 13. Actual and predicted rates of Tier 2 molecular pathology procedures* in Medicare enrollees. Supplementary Figure 14. Actual and predicted rates of any cancer genetic testing* in Medicaid enrollees. Supplementary Figure 15. Actual and predicted rates of any cancer genetic testing* in Commercial plan enrollees. Supplementary Figure 16. Actu [file 13053_2022_226_MOESM1_ESM.docx]

Supplement table 1: Diagnosis codes used to identify mental health disorders

| Conditions | Diagnosis codes |
| --- | --- |
| Nicotine dependence | ICD-9-CM: ‘3051xx’  ICD-10-CM: ‘F17xxx’ |
| Anxiety disorders | ICD-9-CM: '29384', '30000', '30001', '30002', '30009', '30010', '30020', '30021', '30022', '30023', '30029', '3003', '3005', '30089', '3009', '3080', '3081', '3082', '3083', '3084', '3089', '30981', '3130', '3131', '31321', '31322', '3133', '31382', '31383'  ICD-10-CM: 'F064', 'F4000', 'F4001', 'F4002', 'F4010', 'F4011', 'F40210', 'F40218', 'F40220', 'F40228', 'F40230', 'F40231', 'F40232', 'F40233', 'F40240', 'F40241', 'F40242', 'F40243', 'F40248', 'F40290', 'F40291', 'F40298', 'F408', 'F409', 'F410', 'F411', 'F413', 'F418', 'F419', 'F42', 'F422', 'F423', 'F424', 'F428', 'F429', 'F430', 'F4310', 'F4311', 'F4312', 'F488', 'F489', 'R452', 'R453', 'R454', 'R455', 'R456', 'R457', 'R4581', 'R4582', 'R4583', 'R4584' |
| Developmental disorders | ICD-9-CM: '3070', '3079', '31531', '31534', '31535', '31539', '31501', '31502', '31509', '31532', '3155', '3158', '317', '3180', '3181', '3182', '319', '31500', '3151', '3152', '3159', '3154'  ICD-10-CM: 'V400', 'V401', 'F70', 'F71', 'F72', 'F73', 'F78', 'F79', 'F800', 'F801', 'F802', 'F804', 'F8081', 'F8082', 'F8089', 'F809', 'F810', 'F812', 'F8181', 'F8189', 'F819', 'F82', 'F88', 'F89', 'F985', 'R4183', 'R480' |
| Miscellaneous mental health disorders | ICD-9-CM: '30012', '30013', '30014', '30015', '3006', '3071', '30750', '30751', '30752', '30753', '30754', '30759', '30016', '30019', '3060', '3061', '3062', '3063', '3064', '30650', '30652', '30653', '30659', '3066', '3067', '3068', '3069', '3021', '3022', '3023', '3024', '30250', '30251', '30252', '30253', '3026', '30270', '30271', '30272', '30273', '30274', '30275', '30276', '30279', '30281', '30282', '30283', '30284', '30285', '30289', '3029', '30651', '30740', '30741', '30742', '30743', '30744', '30745', '30746', '30747', '30748', '30749', '30011', '3007', '30081', '30082', '30780', '30781', '30789', '29389', '2939', '3101', '316', '64840', '64841', '64842', '64843', '64844', 'V402', 'V403', 'V4031', 'V4039', 'V409', 'V673'  ICD-10-CM: 'F061', 'F068', 'F440', 'F441', 'F442', 'F444', 'F445', 'F446', 'F447', 'F4481', 'F4489', 'F449', 'F450', 'F451', 'F4520', 'F4521', 'F4522' , 'F4529', 'F4541', 'F4542', 'F458', 'F459', 'F481', 'F5000', 'F5001', 'F5002', 'F502', 'F508', 'F5081', 'F5082', 'F5089', 'F509',  'F5101', 'F5102', 'F5103', 'F5104', 'F5105', 'F5109', 'F5111', 'F5112', 'F5113', 'F5119', 'F513', 'F514', 'F515', 'F518', 'F519', 'F520', 'F521', 'F5221', 'F5222', 'F5231', 'F5232', 'F524', 'F525', 'F526', 'F528', 'F529', 'F53', 'F530', 'F531', 'F54', 'F59', 'F640', 'F641', 'F648', 'F649', 'F650', 'F651', 'F652', 'F653', 'F654', 'F6550', 'F6551', 'F6552', 'F6581', 'F6589', 'F659', 'F66', 'F6810', 'F6811', 'F6812', 'F6813', 'F688', 'F68A', 'F99', 'O906', 'R37', 'R4589', 'Z87890', 'Z9183' |

Supplement Figure 1: Sample selection for the exploratory models (2^nd^ objective of the study)

fTest Dataset

(n=747,980)

fTraining Dataset

(n=747,980)

fExclude enrollees with any cancer prior to the genetic tests

(n=1,858)

fExclude enrollees with Age < 10 years (n=280,261)

fAPCD Data

(n=1,778,079)

Supplement Figure 2: Actual and predicted rates of Hereditary Breast and Ovarian Cancer (HBOC) genetic testing in Medicaid enrollees


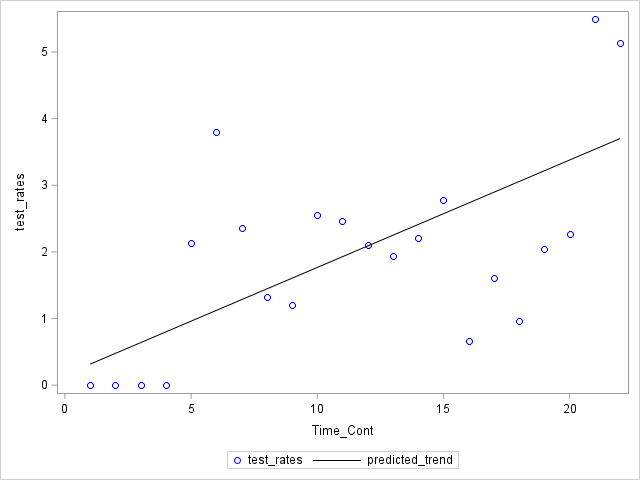


Supplement Figure 3: Actual and predicted rates of Hereditary Breast and Ovarian Cancer (HBOC) genetic testing in Commercial plan enrollees


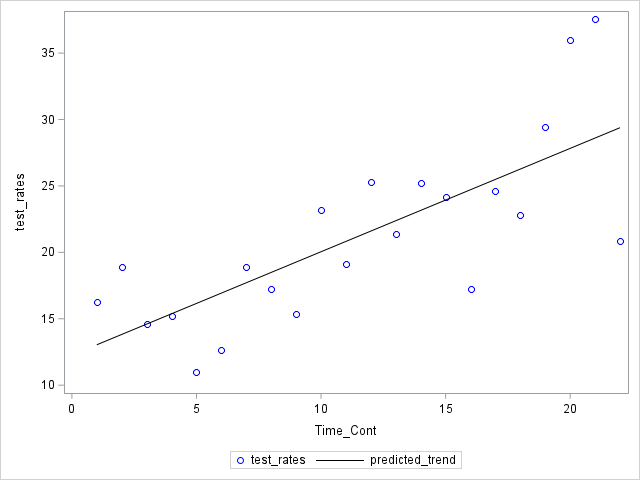


Supplement Figure 4: Actual and predicted rates of Hereditary Breast and Ovarian Cancer (HBOC) genetic testing in State Employee plan enrollees
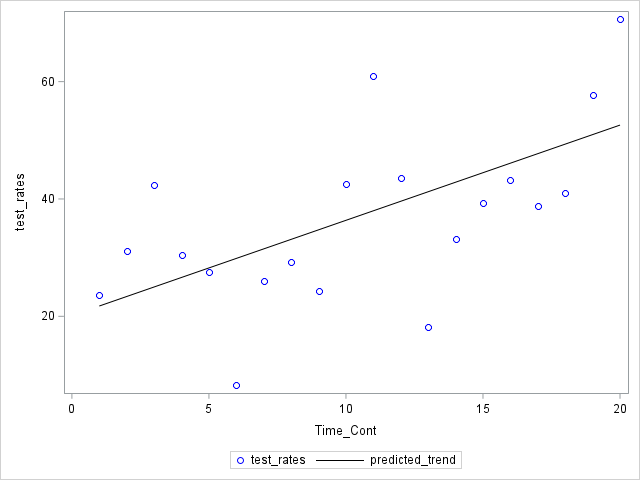


Supplement Figure 5: Actual and predicted rates of Hereditary Breast and Ovarian Cancer (HBOC) genetic testing in Medicare enrollees


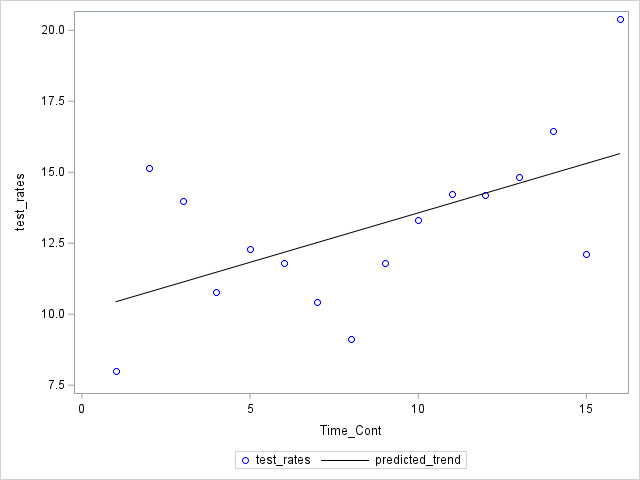


Supplement Figure 6: Actual and predicted rates of Lynch syndrome genetic testing in Medicaid enrollees


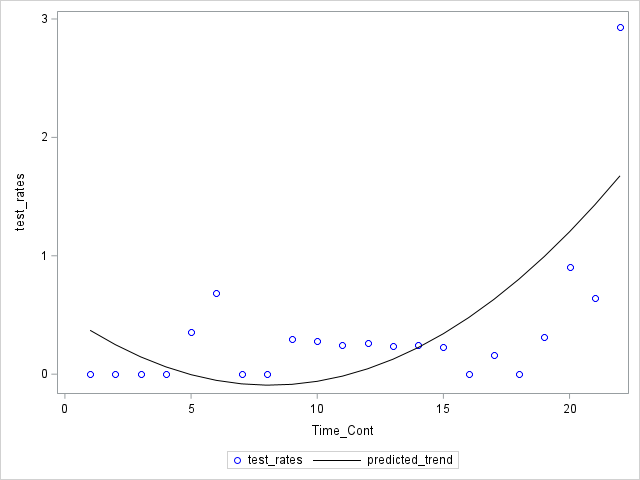


Supplement Figure 7: Actual and predicted rates of Lynch syndrome genetic testing in Commercial enrollees


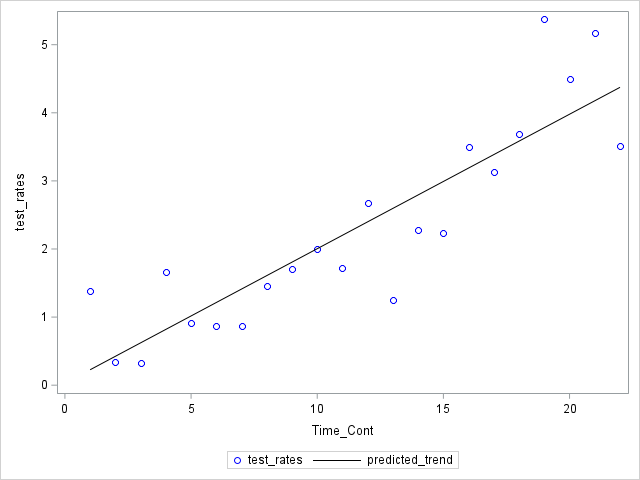


Supplement Figure 8: Actual and predicted rates of Lynch syndrome genetic testing in State employee plan enrollees


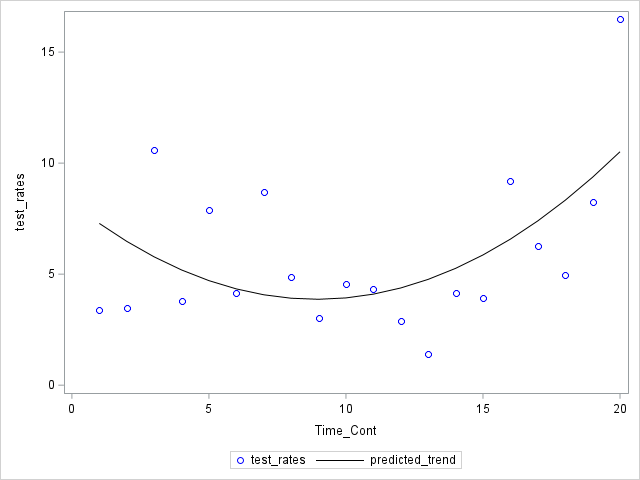


Supplement Figure 9: Actual and predicted rates of Lynch syndrome genetic testing in Medicare enrollees


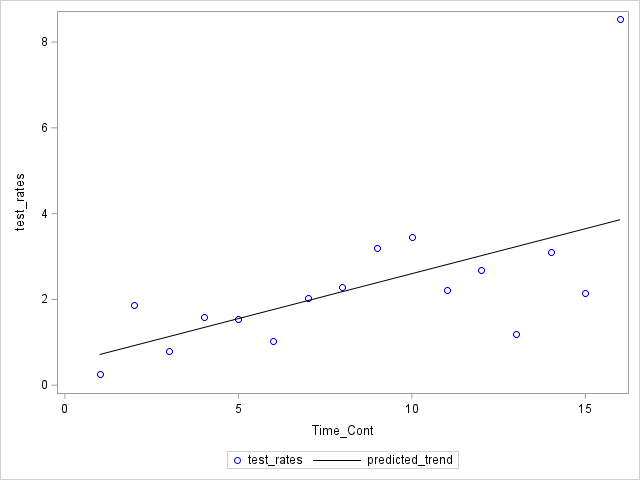


Supplement Figure 10: Actual and predicted rates of Tier 2 molecular pathology procedures * in Medicaid enrollees


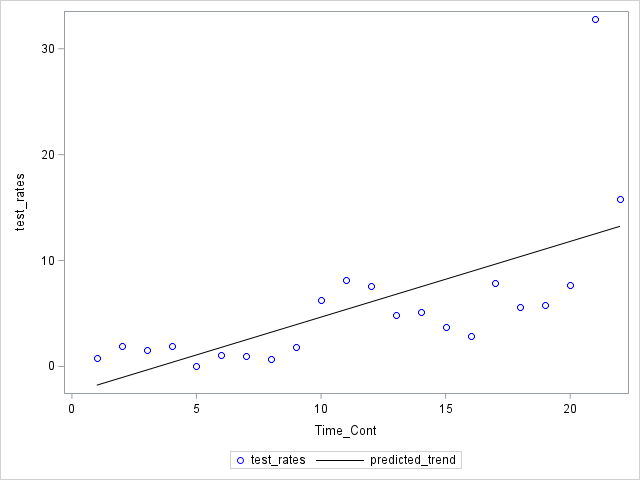


* Tier 2 molecular pathology procedures are based on the complexity of the testing technique and are not specific to a gene or hereditary cancer syndrome.

Supplement Figure 11: Actual and predicted rates of Tier 2 molecular pathology procedures* in Commercial plan enrollees


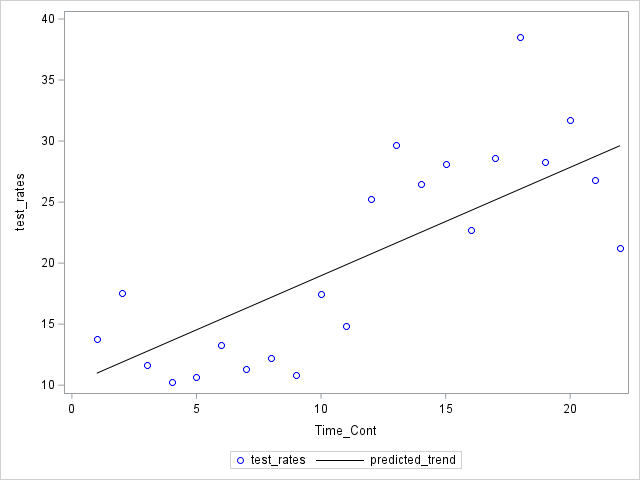


* Tier 2 molecular pathology procedures are based on the complexity of the testing technique and are not specific to a gene or hereditary cancer syndrome.

Supplement Figure 12: Actual and predicted rates of Tier 2 molecular pathology procedures* in State employee plan enrollees


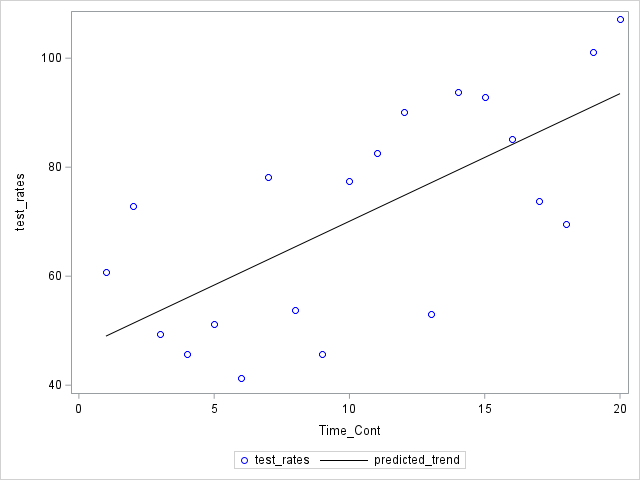


* Tier 2 molecular pathology procedures are based on the complexity of the testing technique and are not specific to a gene or hereditary cancer syndrome.

Supplement Figure 13: Actual and predicted rates of Tier 2 molecular pathology procedures* in Medicare enrollees


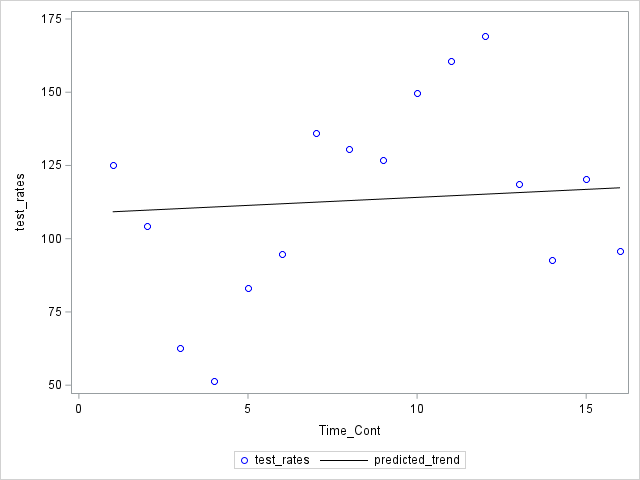


* Tier 2 molecular pathology procedures are based on the complexity of the testing technique and are not specific to a gene or hereditary cancer syndrome.

Supplement Figure 14: Actual and predicted rates of any cancer genetic testing* in Medicaid enrollees


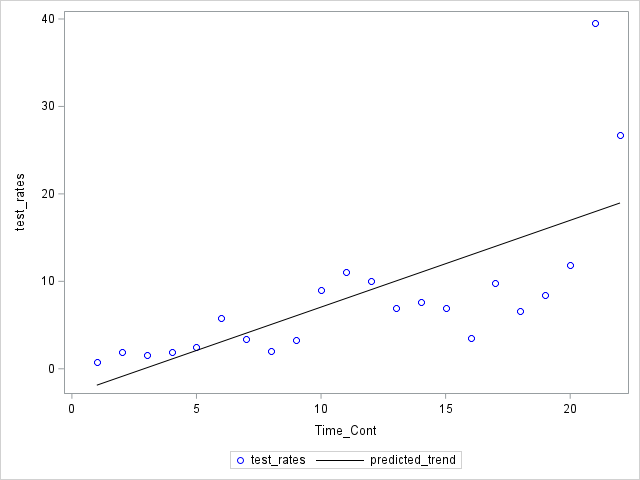


* Any cancer genetic testing included tests in the HBOC, Lynch syndrome, Tier 2 molecular pathology procedures, and other hereditary cancer syndrome (HCS) panel categories.

Supplement Figure 15: Actual and predicted rates of any cancer genetic testing* in Commercial plan enrollees


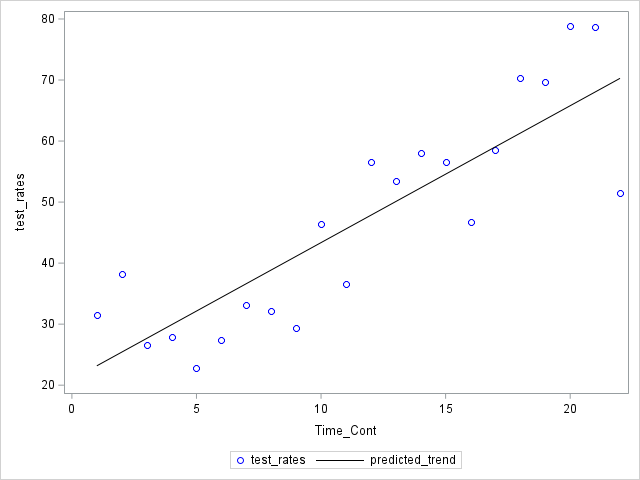


* Any cancer genetic testing included tests in the HBOC, Lynch syndrome, Tier 2 molecular pathology procedures, and other hereditary cancer syndrome (HCS) panel categories.

Supplement Figure 16: Actual and predicted rates of any cancer genetic testing* in State employee plan enrollees


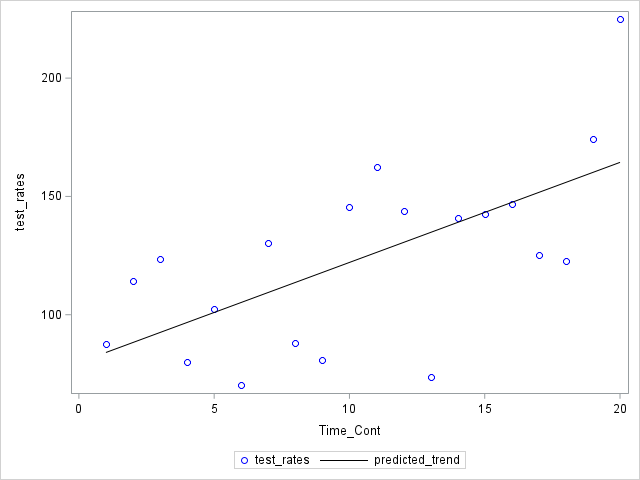


* Any cancer genetic testing included tests in the HBOC, Lynch syndrome, Tier 2 molecular pathology procedures, and other hereditary cancer syndrome (HCS) panel categories.

Supplement Figure 17: Actual and predicted rates of any cancer genetic testing* in Medicare enrollees


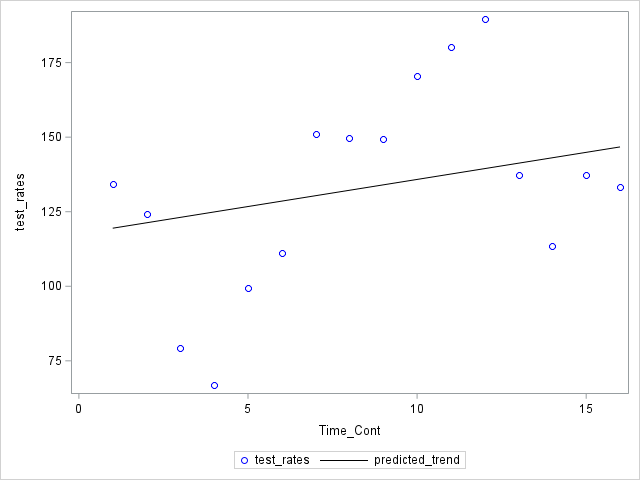


* Any cancer genetic testing included tests in the HBOC, Lynch syndrome, Tier 2 molecular pathology procedures, and other hereditary cancer syndrome (HCS) panel categories.

Supplement Figure 18: Actual and predicted rates of Hereditary Breast and Ovarian Cancer (HBOC) genetic testing in men 18–64 years old


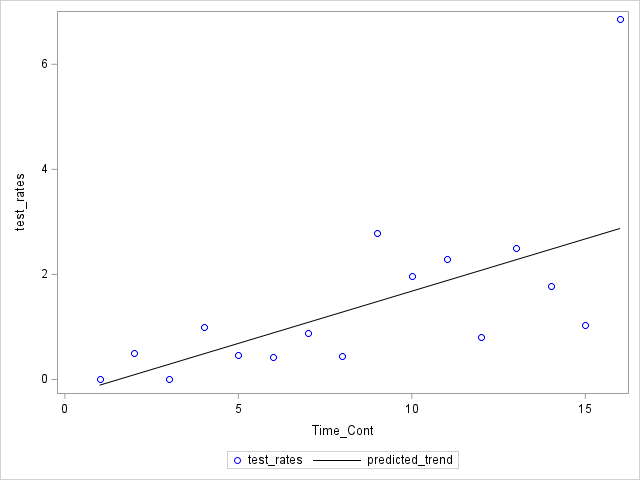


Supplement Figure 19: Actual and predicted rates of Hereditary Breast and Ovarian Cancer (HBOC) genetic testing in men ≥65 years old


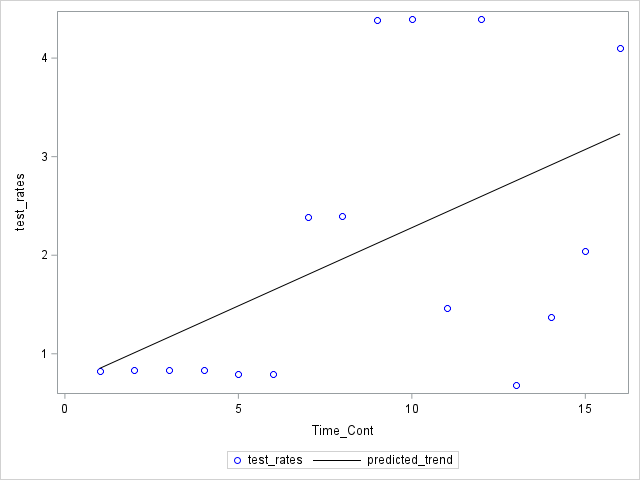


Supplement Figure 20: Actual and predicted rates of Hereditary Breast and Ovarian Cancer (HBOC) genetic testing in women 18–64 years old


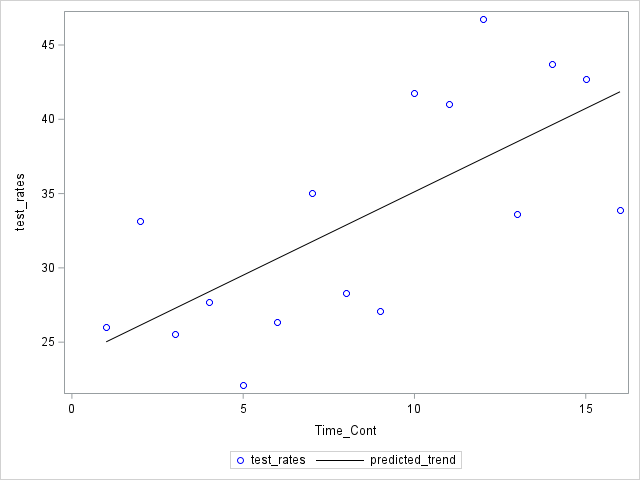


Supplement Figure 21: Actual and predicted rates of Hereditary Breast and Ovarian Cancer (HBOC) genetic testing in women ≥65 years old


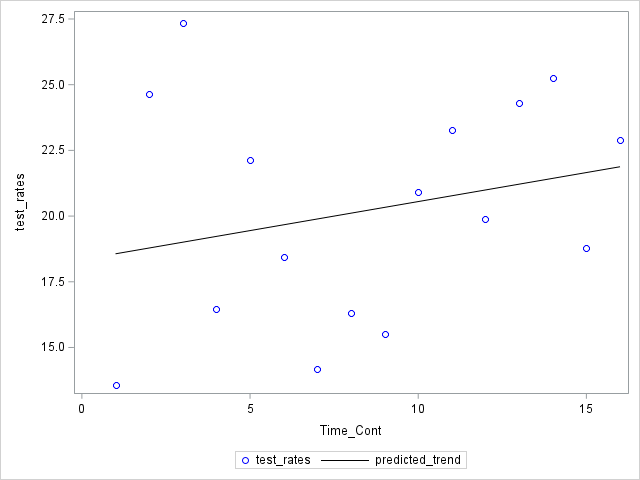


Supplement Figure 22: Actual and predicted rates of Lynch syndrome genetic testing in men 18–64 years old


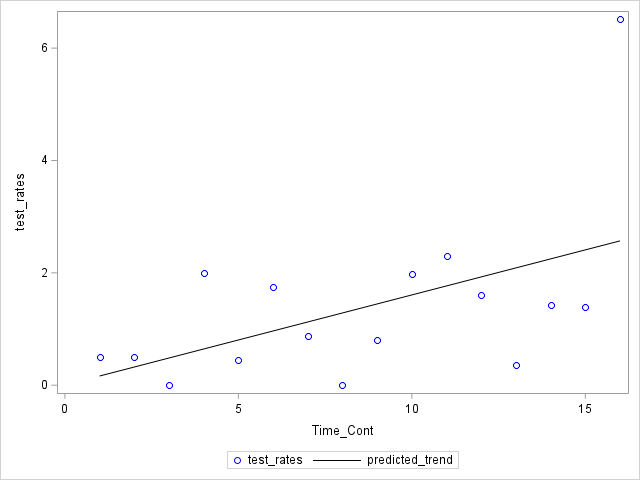


Supplement Figure 23: Actual and predicted rates of Lynch syndrome genetic testing in men

≥65 years old


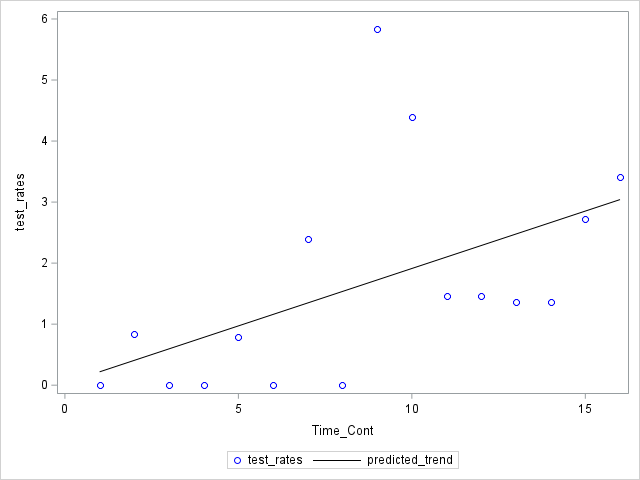


Supplement Figure 24: Actual and predicted rates of Lynch syndrome genetic testing in women 18–64 years old


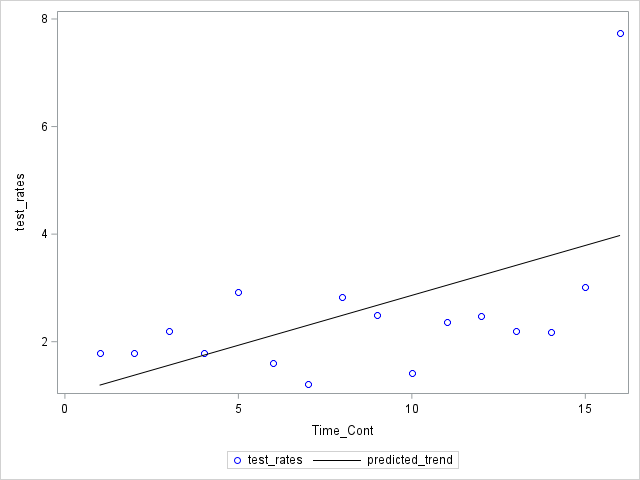


Supplement Figure 25: Actual and predicted rates of Lynch syndrome genetic testing in women ≥65 years old


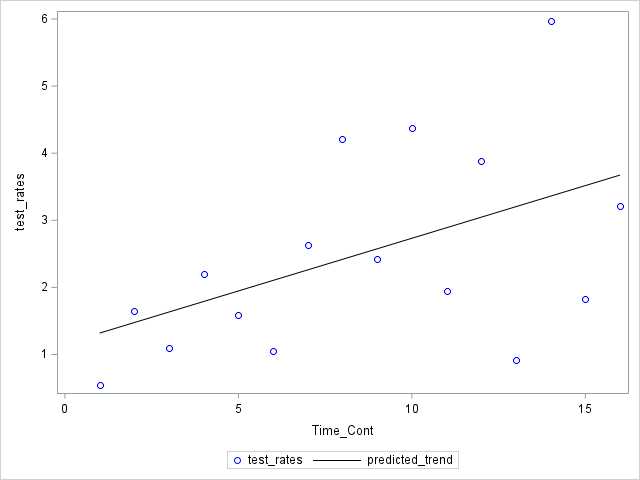


Supplement Figure 26: Actual and predicted rates of Tier 2 molecular pathology procedures* in male enrollees <18 years old


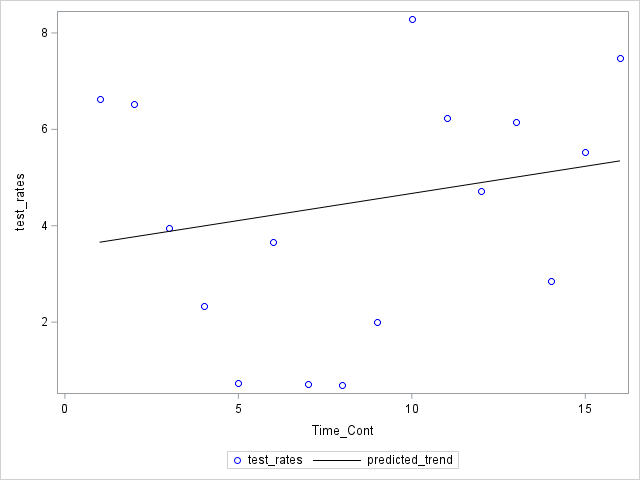


* Tier 2 molecular pathology procedures are based on the complexity of the testing technique and are not specific to a gene or hereditary cancer syndrome.

Supplement Figure 27: Actual and predicted rates of Tier 2 molecular pathology procedures* in men 18–64 years old


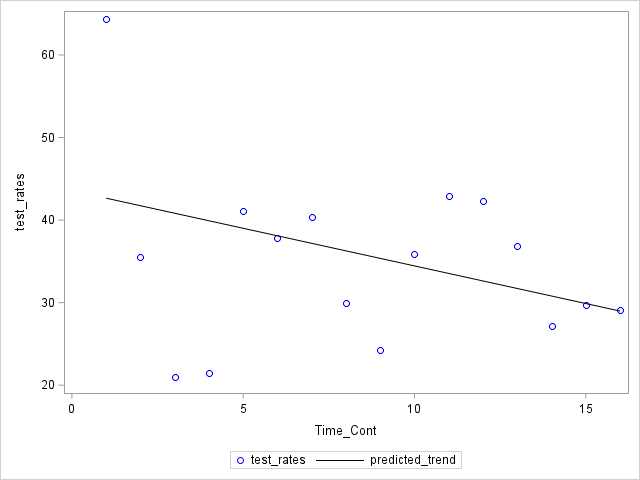


* Tier 2 molecular pathology procedures are based on the complexity of the testing technique and are not specific to a gene or hereditary cancer syndrome.

Supplement Figure 28: Actual and predicted rates of Tier 2 molecular pathology procedures* in men ≥65 years old


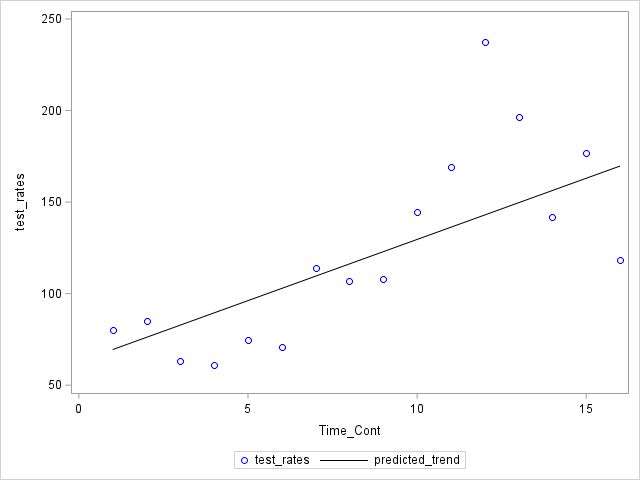


* Tier 2 molecular pathology procedures are based on the complexity of the testing technique and are not specific to a gene or hereditary cancer syndrome.

Supplement Figure 29: Actual and predicted rates of Tier 2 molecular pathology procedures* in female enrollees <18 years old


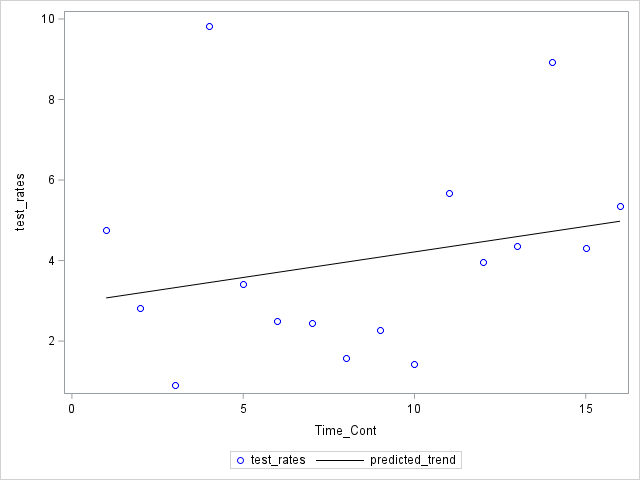


* Tier 2 molecular pathology procedures are based on the complexity of the testing technique and are not specific to a gene or hereditary cancer syndrome.

Supplement Figure 30: Actual and predicted rates of Tier 2 molecular pathology procedures* in women 18–64 years old


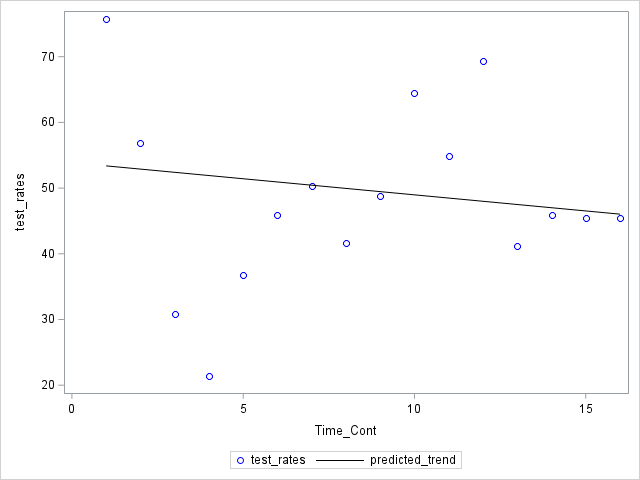


* Tier 2 molecular pathology procedures are based on the complexity of the testing technique and are not specific to a gene or hereditary cancer syndrome.

Supplement Figure 31: Actual and predicted rates of Tier 2 molecular pathology procedures* in women ≥65 years old


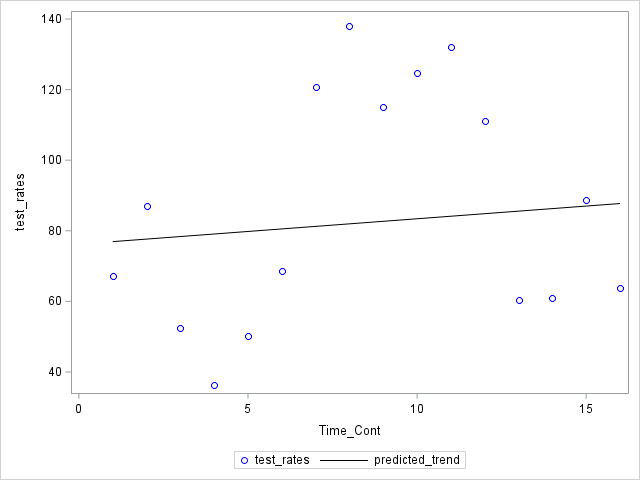


* Tier 2 molecular pathology procedures are based on the complexity of the testing technique and are not specific to a gene or hereditary cancer syndrome.

Supplement Figure 32: Actual and predicted rates of any cancer genetic testing* in male enrollees <18 years old


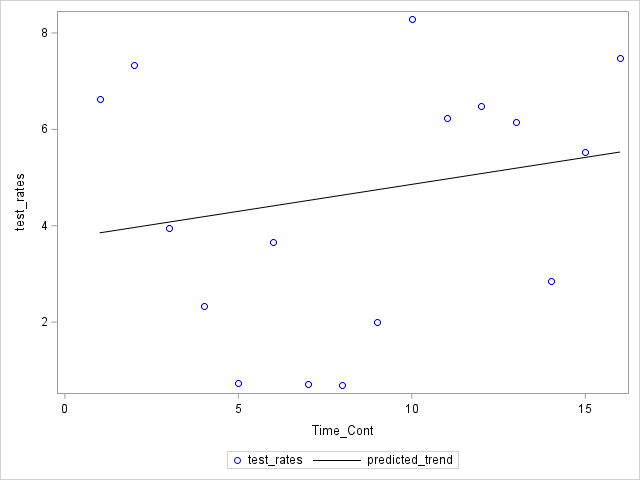


* Any cancer genetic testing included tests in the HBOC, Lynch syndrome, Tier 2 molecular pathology procedures, and other hereditary cancer syndrome (HCS) panel categories.

Supplement Figure 33: Actual and predicted rates of any cancer genetic testing* in men 18–64 years old


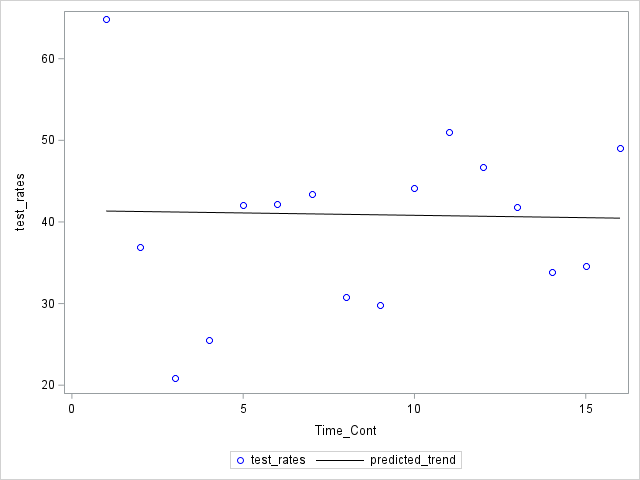


* Any cancer genetic testing included tests in the HBOC, Lynch syndrome, Tier 2 molecular pathology procedures, and other hereditary cancer syndrome (HCS) panel categories.

Supplement Figure 34: Actual and predicted rates of any cancer genetic testing* in men ≥65 years old


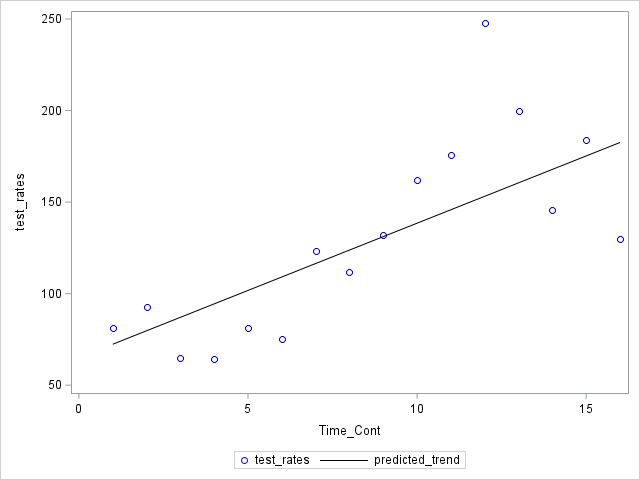


* Any cancer genetic testing included tests in the HBOC, Lynch syndrome, Tier 2 molecular pathology procedures, and other hereditary cancer syndrome (HCS) panel categories.

Supplement Figure 35: Actual and predicted rates of any cancer genetic testing* in female enrollees <18 years old


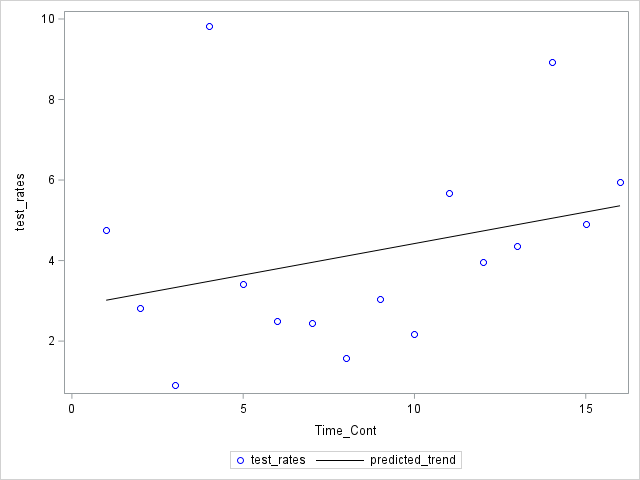


* Any cancer genetic testing included tests in the HBOC, Lynch syndrome, Tier 2 molecular pathology procedures, and other hereditary cancer syndrome (HCS) panel categories.

Supplement Figure 36: Actual and predicted rates of any cancer genetic testing* in women 18–64 years old


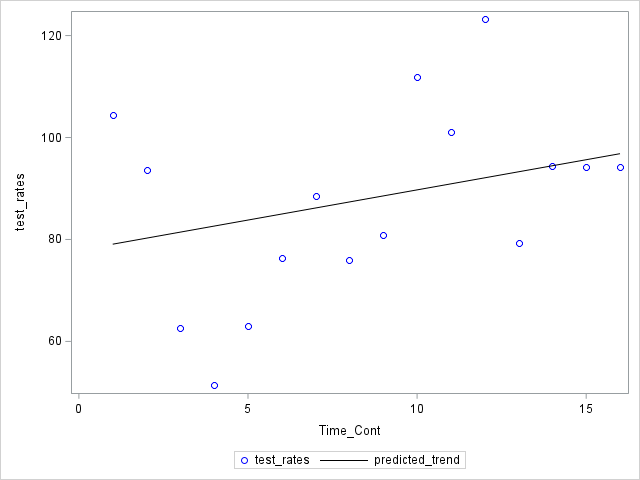


* Any cancer genetic testing included tests in the HBOC, Lynch syndrome, Tier 2 molecular pathology procedures, and other hereditary cancer syndrome (HCS) panel categories.

Supplement Figure 37: Actual and predicted rates of any cancer genetic testing* in women ≥65 years old


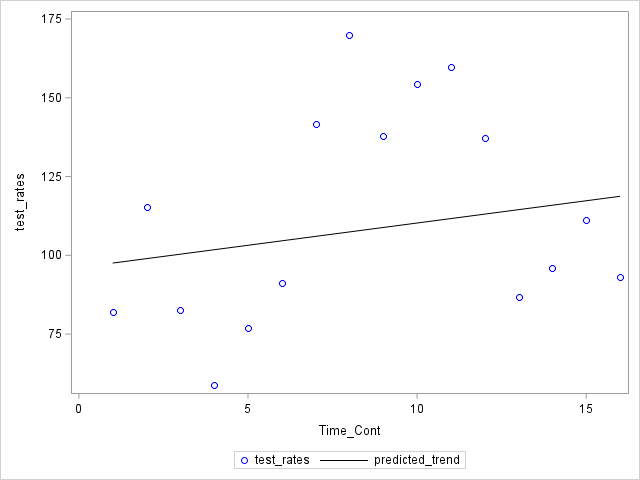


* Any cancer genetic testing included tests in the HBOC, Lynch syndrome, Tier 2 molecular pathology procedures, and other hereditary cancer syndrome (HCS) panel categories.
